# Constructing a Hospital Department Development–Level Assessment Model: Machine Learning and Expert Consultation Approach in Complex Hospital Data Environments

**DOI:** 10.2196/54638

**Published:** 2024-09-04

**Authors:** Jingkun Liu, Jiaojiao Tai, Junying Han, Meng Zhang, Yang Li, Hongjuan Yang, Ziqiang Yan

**Affiliations:** 1 Big Data Analysis Center Honghui Hospital, Xi'an Jiaotong University Xi'an China; 2 School of Foreign Studies Xi'an Medical University Xi'an China

**Keywords:** machine algorithms, hospital management, model construction, support vector machine, clustering

## Abstract

**Background:**

Every hospital manager aims to build harmonious, mutually beneficial, and steady-state departments. Therefore, it is important to explore a hospital department development assessment model based on objective hospital data.

**Objective:**

This study aims to use a novel machine learning algorithm to identify key evaluation indexes for hospital departments, offering insights for strategic planning and resource allocation in hospital management.

**Methods:**

Data related to the development of a hospital department over the past 3 years were extracted from various hospital information systems. The resulting data set was mined using neural machine algorithms to assess the possible role of hospital departments in the development of a hospital. A questionnaire was used to consult senior experts familiar with the hospital to assess the actual work in each hospital department and the impact of each department’s development on overall hospital discipline. We used the results from this questionnaire to verify the accuracy of the departmental risk scores calculated by the machine learning algorithm.

**Results:**

Deep machine learning was performed and modeled on the hospital system training data set. The model successfully leveraged the hospital’s training data set to learn, predict, and evaluate the working and development of hospital departments. A comparison of the questionnaire results with the risk ranking set from the departments machine learning algorithm using the cosine similarity algorithm and Pearson correlation analysis showed a good match. This indicates that the department development assessment model and risk score based on the objective data of hospital systems are relatively accurate and objective.

**Conclusions:**

This study demonstrated that our machine learning algorithm provides an accurate and objective assessment model for hospital department development. The strong alignment of the model's risk assessments with expert opinions, validated through statistical analysis, highlights its reliability and potential to guide strategic hospital management decisions.

## Introduction

The planning and equipping of health care and hospital department systems represent among the most complex tasks [1]. Hospital managers are increasingly concerned with not only developing the hospital’s core competitiveness but also creating a harmonious steady-state ecological environment in a balanced and cooperative way. They recognize that a harmonious environment is a crucial factor in enhancing the hospital’s overall competitiveness, vitality, sustainability, and ability to treat critically ill patients. The use of machine algorithms to construct a sophisticated and practical assessment model for the harmonious development of a hospital department aligns with the principles of realistic management science and hospital operational values. Consequently, hospital administrators are inclined to use independent internal data to construct such a machine algorithm and model. Most studies on machine algorithms in health care focus on clinical research [2], but increased attention is required on how to use the massive amount of hospital data to build hospital management models.

With the improvement and popularization of the technologies of global hospital information, hospital information system (HIS) interconnection, and business intelligence, many hospital systems are generating massive amounts of data with typical big data characteristics. Therefore, exploring deep machine learning algorithms, such as neural networks, to build a hospital department development evaluation model can be significant in guiding hospital managers to reasonably equip and promote the harmonious development of hospital departments and divisions. Currently, machine learning algorithm models are used in various fields to predict research directions [3]. Consensus clustering is a common clustering method used in machine learning algorithms. It uses repeated sampling to draw a certain sample for the data set, specifies the number of *k* clusters, and calculates the rationality under different numbers of clusters. The *k* means algorithm is a widely used clustering analysis algorithm that is simple, efficient, and can make the clustering results locally optimal [4]. The support vector machine (SVM) algorithm is also one of the most popular and discussed machine learning algorithms, which works by constructing a multidimensional hyperplane that optimally differentiates two classes by maximizing the gap between clusters of data [5].

This study uses historical objective data from a specific research hospital, encompassing key indicators of workload, work difficulty, and the diagnosis-related groups (DRG) payment method. The widespread application of the DRG payment method globally bolsters the scientific and universal nature of our research findings. For evaluation, we selected Honghui Hospital, Xi'an Jiaotong University as the subject of our study. By using a series of advanced machine learning algorithms, including cluster analysis, dimensionality reduction, and regression analysis, we conducted an in-depth study and analysis to establish evaluation indicators for hospital departments and to construct an evaluation model. Furthermore, we developed a departmental risk model and conducted cosine similarity comparison analysis and Pearson correlation analysis with departmental development scores obtained from expert surveys. These efforts improved the accuracy and reliability of our model.

## Methods

### Source of Data Sets

The data sets analyzed were derived from those generated by each of the hospitals’ data management systems, namely HIS, the picture archiving and communication system, and the laboratory information system. The data sets span the past 3 years and include various indicators describing workload, work difficulty, drug usage, number of medical tests, and charges for a total of 34 indexes, the specific names of which and their IDs are listed in Multimedia Appendix 1.

### Consensus Clustering

Based on the collected data from 34 indexes, we conducted an unsupervised cluster analysis of hospital departments using the consensus clustering method, with the aim of categorizing these departments into groups with distinct characteristics. This clustering process was facilitated using the R *ConsensusClusterPlus* package (R Foundation for Statistical Computing) [6], with parameters set to 50 repetitions and a resampling rate of 80% (pItem=0.8) to enhance the robustness of the analysis. Furthermore, to validate the effectiveness and reliability of the clustering results, we performed a principal component analysis on the 34 indexes of the hospital departments and used the data visualization tool R package *ggplot2* for an intuitive graphical representation of the findings.

### K Means Clustering Algorithm Used for Clustering Hospital Departments

To validate the results of consensus clustering, we used the *k* means clustering algorithm to recluster the hospital departments. For a comprehensive feature selection process, we also used the SVM recursive feature elimination algorithm to sift through 34 potential indicators, identifying key features that significantly impact hospital development. This selection process is implemented using the *e1071* package [7].

To verify that the filtered feature indexes were representative, we again clustered the hospital departments using the *k* means clustering algorithm and these feature indexes to determine if the new clusters were consistent with the hospital department classification results of the unsupervised clustering method. Detailed introductions to the *k* means clustering algorithm and the SVM recursive feature algorithm can be found in Multimedia Appendix 2.

### Characteristic Indexes Model Construction

Based on the feature indexes, we constructed a logistic regression prediction model using the R language. Using this model, we developed a nomogram designed to predict risk scores for various departments. The departments were then assessed and ranked according to these predicted risk scores. Furthermore, to ensure the predictive accuracy of the model, a calibration curve was applied to rigorously validate the goodness-of-fit of the model.

### Correlation Analysis of Hospital Departments

To investigate the direct or indirect network interactions between different clustered departments within the hospital, we first conducted a Spearman correlation analysis on the data matrix of the 2 clustered departments, with a screening criterion of cor>0.4 and *P*<.05. Additionally, to construct an interoperability network between the departments, we used Cytoscape software (version 3.9.1, The Cytoscape Consortium). To gain further insight into the potential interaction coefficients between the components and different clustered departments, we performed a correlation analysis using the *corrplot* package in R. The outcomes were presented in the form of a heat map.

### Questionnaire and Cosine Similarity Verification

The questionnaires included the experts’ sex, age, profession, years of experience, and title. Most experts had more than 20 years of work experience (n=23, 68%) and were largely chief physicians or associate chief physicians (n=31, 82%). A total of 34 valid questionnaires were used in our analysis, providing valuable insights into the hospital departments’ development. Multimedia Appendix 2 delineates the scoring criteria and elucidates the methodology for the computation of weighted scores. Ultimately, we used the cosine similarity algorithm to ascertain the congruence between the rankings of overall departmental development scores and the risk rankings assigned by the machine algorithm to the departments. Additionally, we applied Pearson correlation analysis to further validate the similarity between these 2 sets of rankings

### Ethical Considerations

The study was conducted in accordance with the Declaration of Helsinki and Declaration of Geneva and was approved by the Ethics Committee of Honghui Hospital, Xi'an Jiaotong University (202209003). Informed consent was obtained from all experts participating in the study. All data used in this study were anonymized to ensure the privacy and confidentiality of the participants. No compensation was provided to the participants for their involvement in this study.

## Results

### Overall Flow

**Figure 1:**
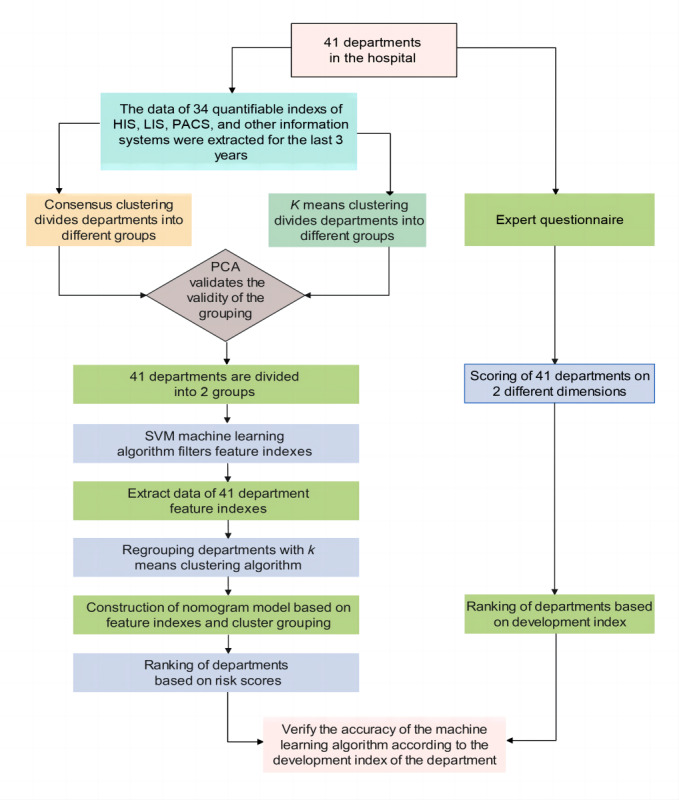
Flow chart of the study procedure. HIS: hospital information system; LIS: laboratory information system; PACS: picture archiving and communication system; PCA: principal component analysis; SVM: support vector machine.

The schematic flow of this study is shown in Figure 1. We collected data on quantifiable indicators from various departments within the hospital's information systems for the past 3 years. The consensus clustering and *K* means clustering algorithms were sequentially applied to categorize the hospital’s 41 departments, with consistent clustering results observed between the 2 methods. Subsequently, the SVM machine learning algorithm was applied to filter the feature indexes and recluster the departments to confirm the representativeness of the selected indexes. Thereafter, we visualized the clustering outcomes and model feature indexes using a nomogram and obtained a risk score and ranking for each department. Additionally, an expert questionnaire was used to score the level and breadth of disciplinary development and the balance of the departments, leading to a composite development score and ranking for the 41 departments. Ultimately, the cosine similarity algorithm and Pearson correlation analysis were jointly applied to validate the similarity between the overall departmental development score ranking and the department risk ranking as determined by the machine algorithm.

### Consensus Clustering and Dividing the Departments Into 2 Clusters

**Figure 2:**
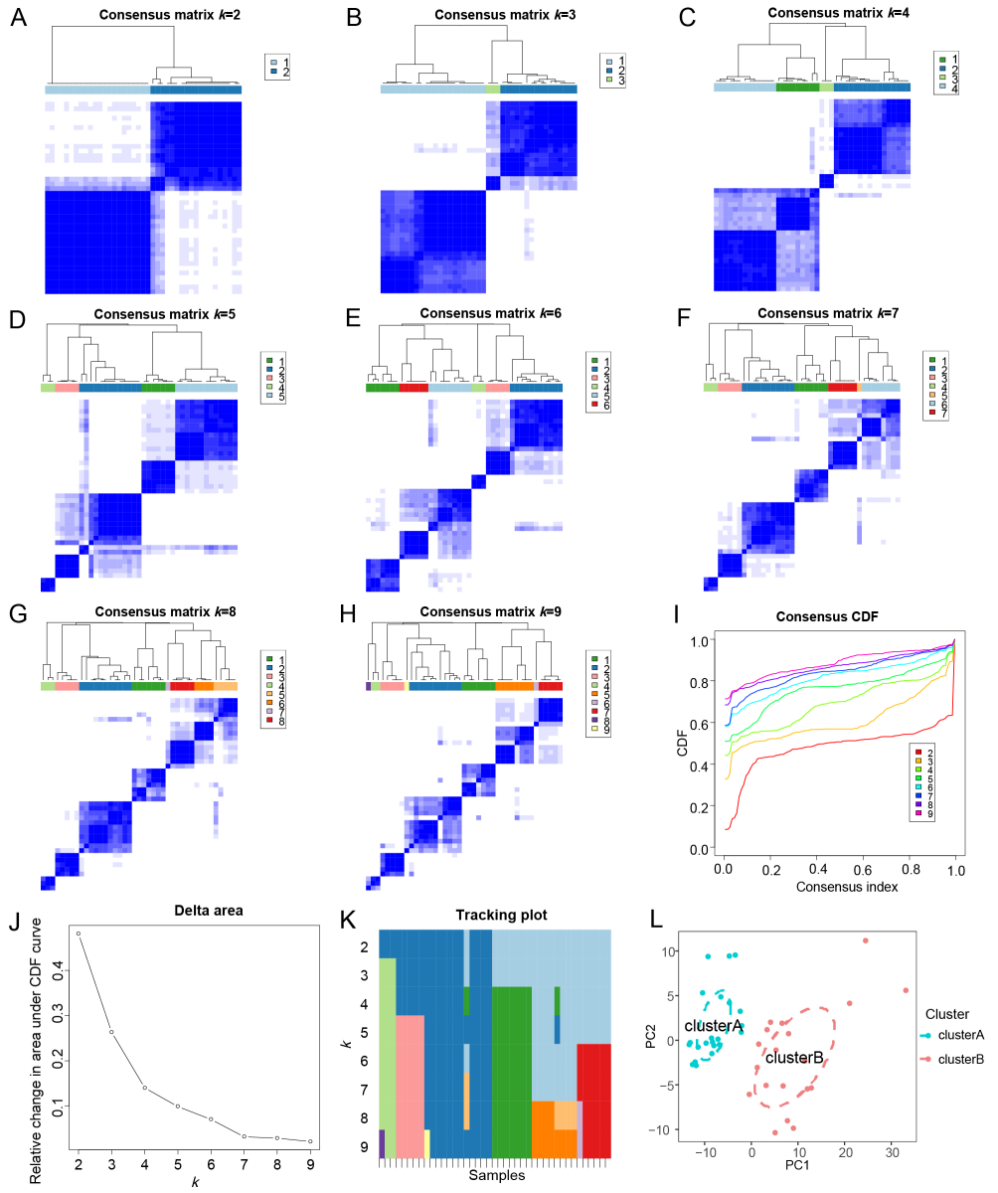
Consensus clustering analysis of hospital departments: heat maps and stability assessments. (A-H) Matrix heat map. The rows and columns of the matrix heat map represent the samples. When k=2, the matrix heat map is clearly separated. The consensus matrix has values ranging from 0=not likely to cluster together to 1=always cluster together, from white to dark blue. The consensus matrix is arranged by consensus classification (tree above the heat map). The bars between the tree view and the heat map represent the categories. (I) The CDF curve. It shows the CDF for different k values, which can be used to determine how to take k, the CDF reaches an approximate maximum, and the cluster analysis results are the most reliable. (J) Delta area score of the CDF curve. (K) Tracking plot of cluster classification. The black stripes at the bottom of the trajectory graph for k=2-9 represent samples, indicating the classification of samples when different k values are taken, and the color blocks of different colors represent different classifications. (L) Principal component analysis of 41 partial indexes. Cluster A is represented in green and cluster B is depicted in red. CDF: cumulative distribution function; PCA: principal component analysis.

The consensus clustering analysis among different departments revealed that the optimal effect for the samples was achieved when the clustering variable (*k*) was equal to 2. The detailed process and results of this analysis were provided in Multimedia Appendix 2. The matrix heat map and cumulative distribution function curve, as depicted in Figure 2, further confirmed the clear separation and stability of the 2 clusters. The departments were robustly divided into cluster A with 22 departments and cluster B with 19 departments, as visualized by the principal component analysis results in Figure 2L.

### Clustering of Departments Using the K Means Clustering Algorithm

**Figure 3:**
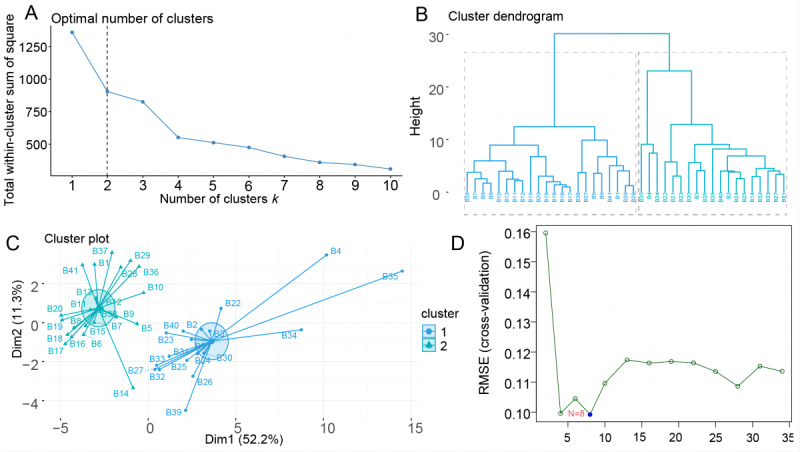
Application of k means clustering and SVM model for index analysis in hospital departments. (A-C) K means clustering analysis. (A) The number k of department types depends on the minimum number of clusters that produce the most meaningful cluster profiles according to the within-cluster sum of squares method (“elbow” method). (B) Cluster dendrogram. The x-axis represents the cross-section and the y-axis represents height. A dendrogram visualizing the order and distances of subjects for merges during hierarchical clustering using the Ward method, with the overlaid colors representing the 2-cluster solution as the optimal k. (C) Scatter plot of the k means clustering results. Cluster A is depicted in green and cluster B in blue. (D) SVM model visualization. The x-axis in the figure indicates the number of extracted key indexes, and the y-axis is the root-mean-square error. The value of the key indexes and their number can be determined by calculating the value of the root mean square error. RMSE: root mean square error; SVM: support vector machine.

To observe the reliability of the consensus clustering results, we used the *k* means clustering algorithm to cluster the 41 departments using 34 indicators. Multimedia Appendix 3 shows the 41-department ID correspondence. We first used the elbow method (Figure 3A) to determine the number of clusters and found that the decline decreased when *k*=2. To observe the similarity of the subclusters from different perspectives, we used a clustering dendrogram to observe the hierarchy of the different subclusters (Figure 3B). Clustering is meaningful when departments are divided into 2 clusters (cluster 1: n=22; cluster 2: n=19). The centroid diagram (Figure 3C) shows that the departments can be clearly divided into 2 clusters, with each department being closest to the corresponding centroid. The *k* means clustering results were consistent with the consensus clustering results.

### Reclustering and Grouping of 41 Hospital Departments Using Indexes Modeled on the SVM Algorithm

**Figure 4:**
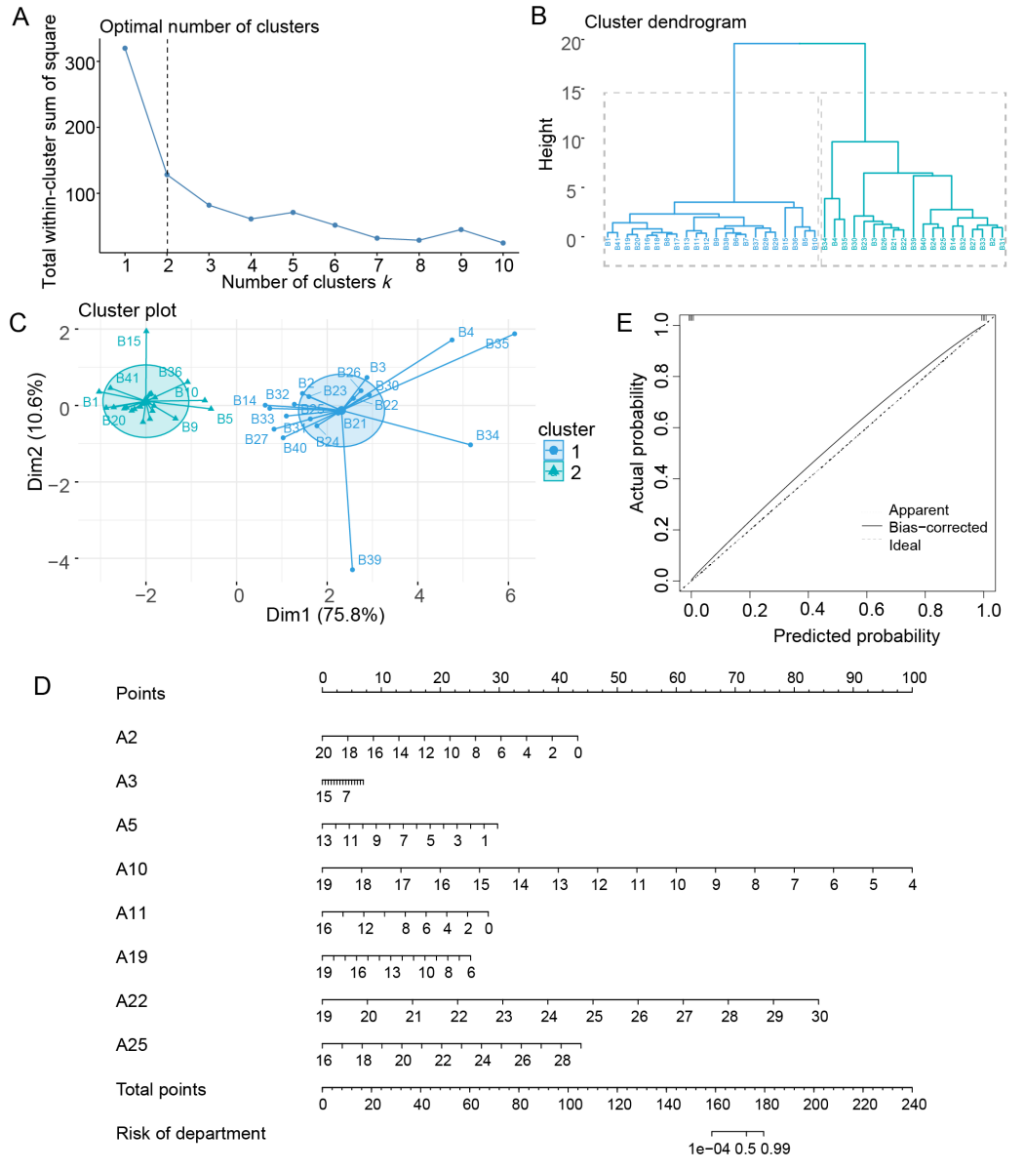
The 8 key indexes k means clustering and nomogram validation. (A-C) K means clustering analysis. (A) The number k of department types depends on the minimum number of clusters that produce the most meaningful cluster profiles according to the within-cluster sum of squares method (“elbow” method). (B) Clustering dendrogram. The x-axis represents the cross-section and the y-axis represents height. Dendrogram visualizes the order and distances of subjects for merges during hierarchical clustering using the Ward method, with the overlaid colors representing the 2-cluster solution as the optimal k. (C) Scatter plot of the k means clustering results. Cluster A is depicted in green and cluster B in blue. (D) Nomogram based on 8 key indexes. A2, A3, A5, A10, A11, A19, A22, and A25 on the left side of the graph represent the 8 key indexes of outpatient visits, number of surgical cases, number of ambulatory (day) surgery number of radiological examinations, number of intraoperative radiological examinations, number of large medical equipment examinations, medical income, and total relative weights, respectively. (E) Calibration curve.

A round-robin algorithm was applied to the SVM model (Figure 3D), and the lowest error rate (0.09) was identified for 8 indexes (outpatient visits, medical income, total relative weights [RW], and number of surgical cases, ambulatory [day] surgery, radiological examinations, intraoperative radiological examinations, and large medical equipment examinations) in the cross-validation. The reclustering analysis using these indexes, as shown in Figures 4A-4C, confirmed the division into 2 clusters, consistent with previous findings.

### Nomogram Model Construction and Validation

**Figure 5:**
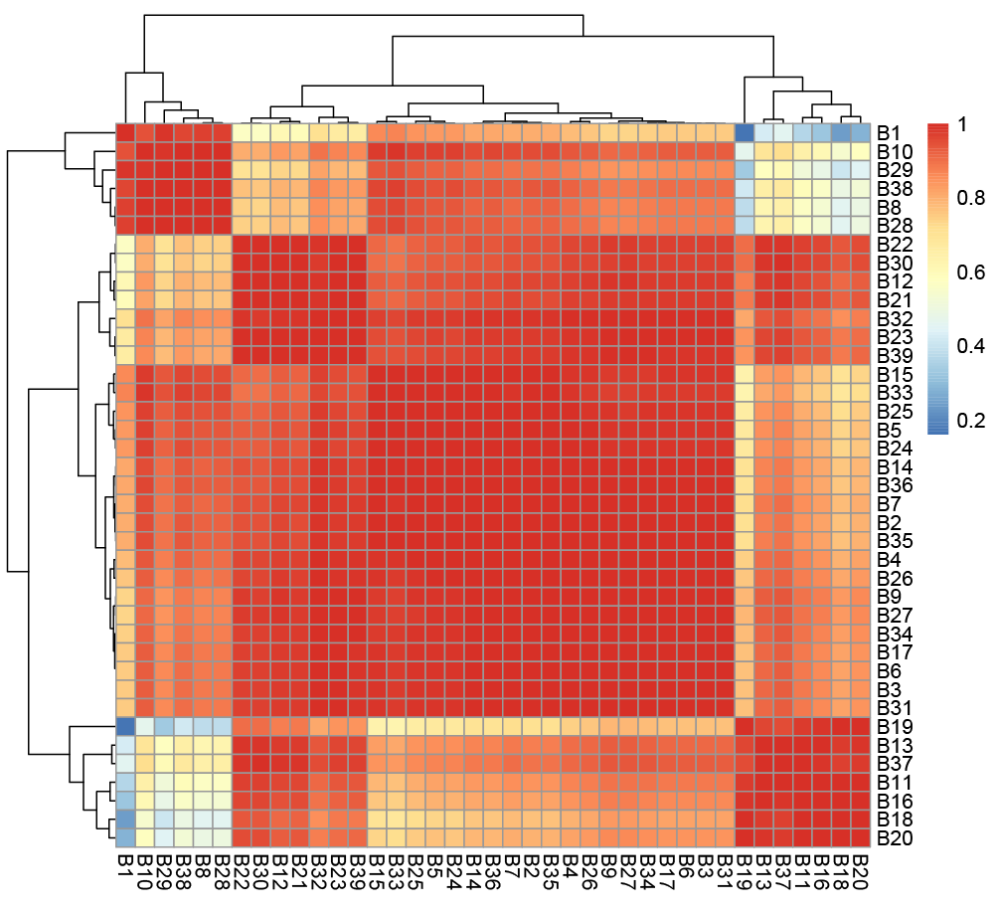
Heat map of cosine similarity between machine learning-derived departmental risk rankings and expert-surveyed development rankings. Red indicates high similarity, and blue indicates low similarity. B1-B41 respectively for the following departments: pediatric orthopedic, ear, nose & throat and head & neck-usually and plastic surgery, rheumatology immunology and endocrinology, gynecology and obstetrics, orthopedic oncology, peripelvic traumatic orthopedic, upper extremity traumatic orthopedic, lower extremity traumatic orthopedic, orthopedic microsurgery, rheumatic and immune-related orthopedic joint surgery, osteonecrosis and joint reconstruction surgery, hip joint surgery, knee joint surgery, respiratory diseases, emergency, cervical surgery, spinal degenerative diseases and spinal oncologic, spinal minimally invasive surgery, lumbar surgery, intervertebral disc diseases and spinal deformities surgery, bone and joint rehabilitation, neurospinal rehabilitation, pain intervention, urology surgery, general practice, neurology, neurosurgery, hand surgery center II, hand surgery center I, gastroenterology, digestive surgery, cardiovascular medicine, thoracic surgery, hematology and oncology, ophthalmology, shoulder and elbow surgery, knee and ankle surgery, integrated traditional Chinese medicine and western medicine orthopedics, critical care medicine, peripheral vascular medicine, and foot and ankle surgery.

Based on the 8 key indexes, we constructed a bar graph model (Figure 4D), and the calibration curve (Figure 4E) showed that the nomogram had strong predictive power. The 11 departments with higher risk scores were as follows: critical care medicine, hematology and oncology, gastroenterology, ophthalmology, gynecology and obstetrics, neurospinal rehabilitation, neurosurgery, neurology, bone and joint rehabilitation, cardiovascular medicine, and pain intervention (Multimedia Appendix 4). Interestingly, the departments with high-risk scores were all departments within cluster B, while the departments with low-risk scores were all within cluster A. Furthermore, the department development scores, which were calculated based on the expert questionnaire scores for 41 departments, demonstrated that departments with high development scores had relatively low-risk scores, whereas departments with low development scores had relatively high-risk scores (Multimedia Appendices 4 and 5). The results of cosine similarity demonstrate a high degree of consistency between the two algorithms, with a similarity coefficient of 0.94 (Figure 5). Pearson correlation analysis revealed a significant correlation between the 2 data sets (correlation coefficient=0.734; *P*=4.85610^–8^).

### Department Network Analysis

**Figure 6:**
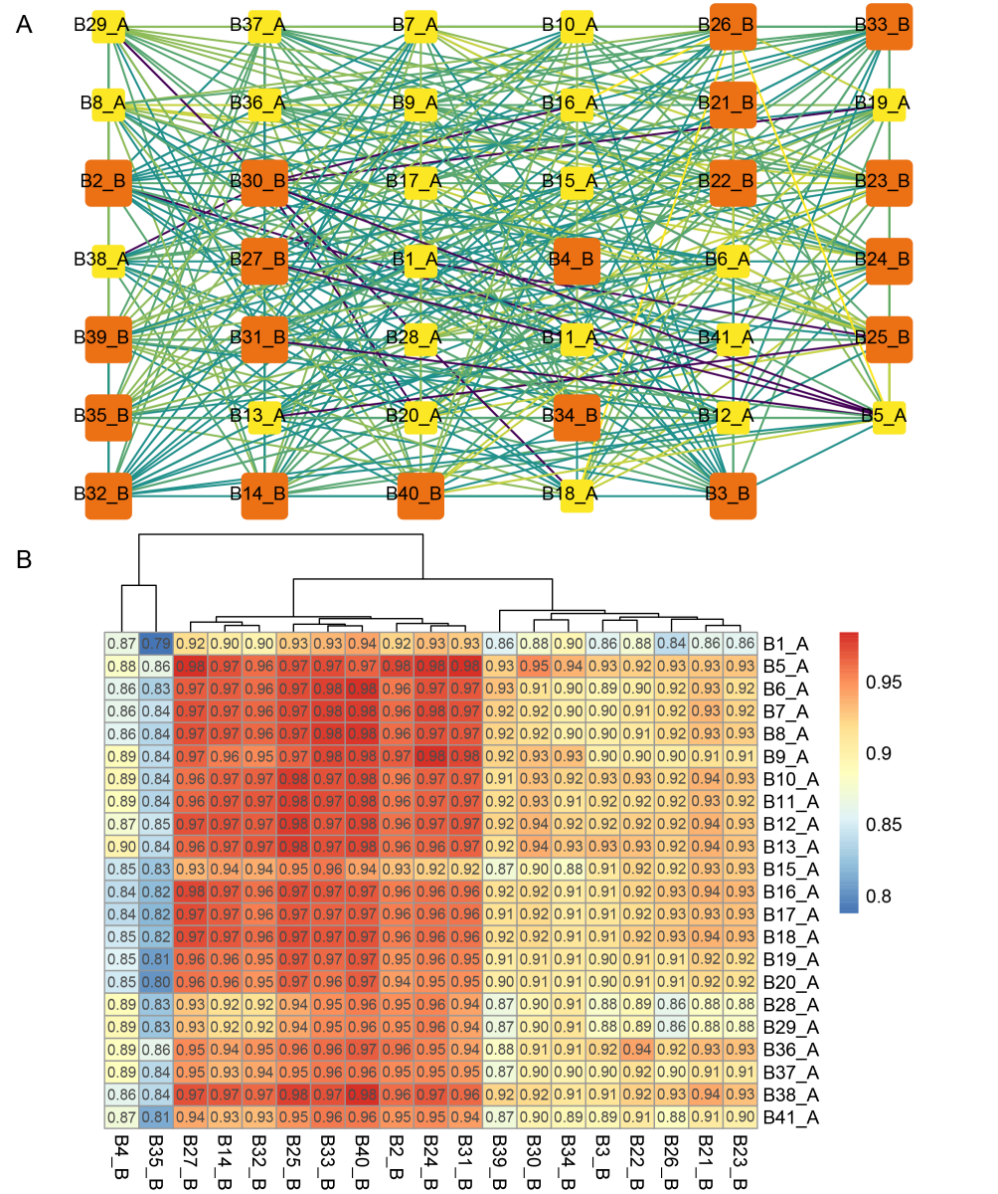
Interaction of different clusters of hospital departments. (A) Network relationship diagram of department interactions. The squares represent the IDs of different departments; yellow is part of cluster A and orange is part of cluster B. The color of the line indicates the correlation coefficient, the lighter the color, the lower the correlation. (B) Heat map of the correlation between the 2 clusters A and B. The more orange the color, the higher the correlation, and the bluer, the lower the correlation. B1-B41 respectively for the following departments: pediatric orthopedic, ear, nose & throat and head & neck-usually and plastic surgery, rheumatology immunology and endocrinology, gynecology and obstetrics, orthopedic oncology, peripelvic traumatic orthopedic, upper extremity traumatic orthopedic, lower extremity traumatic orthopedic, orthopedic microsurgery, rheumatic and immune-related orthopedic joint surgery, osteonecrosis and joint reconstruction surgery, hip joint surgery, knee joint surgery, respiratory diseases, emergency, cervical surgery, spinal degenerative diseases and spinal oncologic, spinal minimally invasive surgery, lumbar surgery, intervertebral disc diseases and spinal deformities surgery, bone and joint rehabilitation, neurospinal rehabilitation, pain intervention, urology surgery, general practice, neurology, neurosurgery, hand surgery center II, hand surgery center I, gastroenterology, digestive surgery, cardiovascular medicine, thoracic surgery, hematology and oncology, ophthalmology, shoulder and elbow surgery, knee and ankle surgery, integrated traditional Chinese medicine and western medicine orthopedics, critical care medicine, peripheral vascular medicine, and foot and ankle surgery.

**Figure 7:**
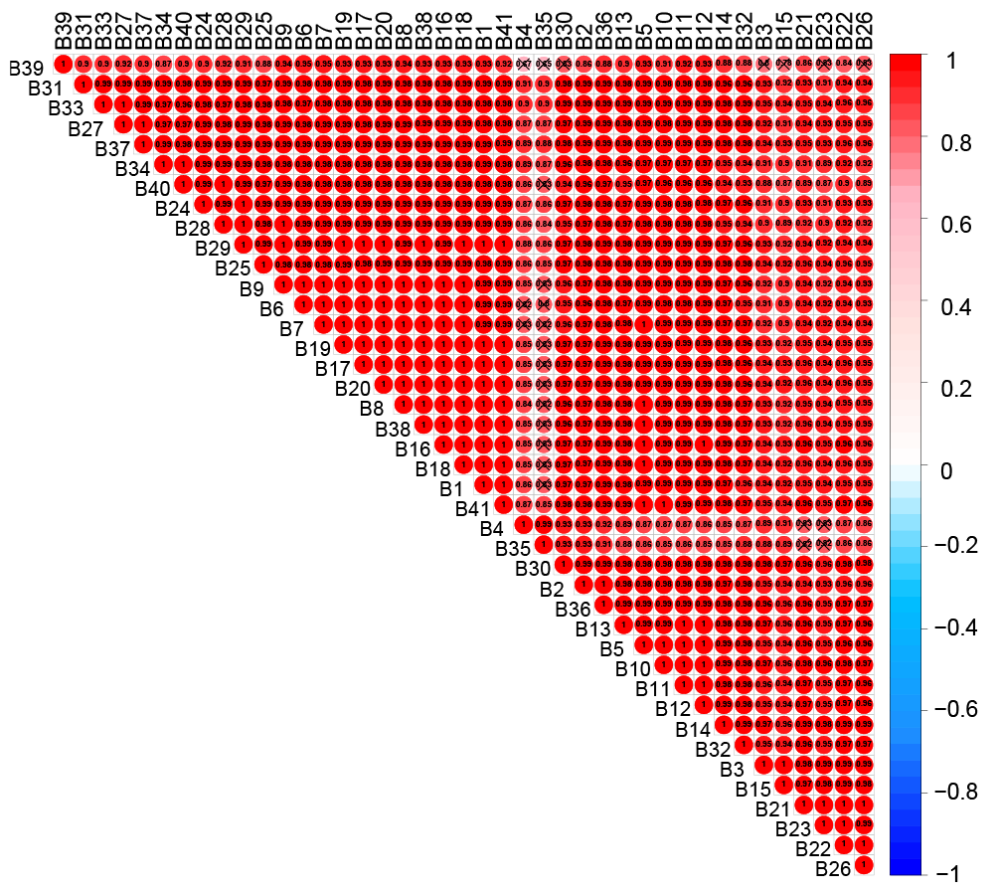
Heat map of correlation analysis for all hospital departments based on 8 key indexes. Red indicates a positive correlation and blue indicates a negative correlation. X indicates a correlation coefficient below 0.85. B1-B41 respectively for the following departments: pediatric orthopedic, ear, nose & throat and head & neck-usually and plastic surgery, rheumatology immunology and endocrinology, gynecology and obstetrics, orthopedic oncology, peripelvic traumatic orthopedic, upper extremity traumatic orthopedic, lower extremity traumatic orthopedic, orthopedic microsurgery, rheumatic and immune-related orthopedic joint surgery, osteonecrosis and joint reconstruction surgery, hip joint surgery, knee joint surgery, respiratory diseases, emergency, cervical surgery, spinal degenerative diseases and spinal oncologic, spinal minimally invasive surgery, lumbar surgery, intervertebral disc diseases and spinal deformities surgery, bone and joint rehabilitation, neurospinal rehabilitation, pain intervention, urology surgery, general practice, neurology, neurosurgery, hand surgery center II, hand surgery center I, gastroenterology, digestive surgery, cardiovascular medicine, thoracic surgery, hematology and oncology, ophthalmology, shoulder and elbow surgery, knee and ankle surgery, integrated traditional Chinese medicine and western medicine orthopedics, critical care medicine, peripheral vascular medicine, and foot and ankle surgery.

The correlation network diagram and heat map (Figure 6) showed that the cluster B departments of respiratory diseases, urology surgery, general practice, neurosurgery, gastroenterology, cardiovascular medicine, thoracic surgery, and peripheral vascular medicine were strongly correlated with almost all departments in cluster A (cor≥0.94). In contrast, the departments of pediatric orthopedic and emergency appeared to be more independent, which may be related to the departmental characteristics or patient populations. The following department pairs showed the strongest correlation: department of orthopedic oncology and department of digestive surgery; department of orthopedic oncology and department of neurology; department of peripheral vascular medicine and department of integrated traditional Chinese medicine and western medicine orthopedics; and department of urology surgery and department of orthopedic microsurgery. Their correlation coefficients are 0.9849, 0.9843, 0.9841, and 0.9840, respectively. Figure 7 shows that the correlation coefficients for the following departments were all greater than 0.998: department of cervical surgery and department of spinal degenerative diseases and spinal oncologic; department of cervical surgery and department of spinal minimally invasive surgery; hand surgery center II and hand surgery center I; department of spinal degenerative diseases and spinal oncologic and department of spinal minimally invasive surgery; department of lumbar surgery and department of intervertebral disc diseases and spinal deformities surgery. Ophthalmology had a lower correlation with 15 departments (correlation coefficient less than 0.85), and gynecology and obstetrics had a lower correlation with 5 departments (correlation coefficient less than 0.85).

## Discussion

### Principal Findings

Many countries now recognize health information technology as a national strategy for the health sector. For example, the United States has enhanced health information technology as part of its health care reform strategy [8]. In China, hospitals above the second level have completed their HIS, laboratory information system, picture archiving and communication system, and other information systems to realize the information and digital foundation of patient services and hospital management [9]. Increasing information technology use in hospitals worldwide has enabled most medical institutions to extract a large amount of data for machine algorithms and to build hospital department evaluation models. These findings can be used to objectively evaluate the development status of hospital departments and determine whether some are in a mature state and will be further differentiated into subspecialty departments. This model also allows us to assess the poor development of certain departments, and hospital administrators should pay more attention to these departments and strengthen their support in all areas.

Under the condition that the model has sufficient complexity and accuracy, the larger the data set, the more suitable the machine learning model is, in theory, for a perfect function. We collected the available data on all 34 fields in each of the hospital's systems, with data sets spanning 3 years. We applied consensus clustering and *k* means algorithms to classify the 41 departments of the study hospital, and the clustering was surprisingly consistent. One cluster included all orthopedic departments of the study hospital. Orthopedics is part of traditional surgery, and some traditional surgical departments in the hospital studies were not clustered into the category that included orthopedics, such as departments of neurosurgery, thoracic surgery, digestive surgery, and urology surgery belonging to a different cluster from all orthopedic departments. This could be because the study hospital is affiliated with a Red Cross institution. The hospital was established in 1911 when China was at war, and the main medical mission of the hospital at its inception was to treat patients with traumatic injuries. Over more than 100 years, the hospital has grown to maintain its traditional strengths in treating trauma patients; however, it is now centrally located in a major city in China (with an urban population of approximately 10 million) and exists in peacetime. Hospital administrators and local governments are increasingly concerned about the comprehensive capacity of hospitals to treat critically ill patients owing to the needs of those in surrounding communities and regions. While the study hospital is gradually developing into a multidisciplinary general hospital, it still has distinctive departmental characteristics (with 1600 beds, 1000 of which are orthopedic), and its orthopedic discipline development has a certain influence in the Chinese region. For 4 consecutive years, the study hospital was ranked among the top 100 hospitals in China (including more than 2000 tertiary hospitals) in the “performance assessment ranking of tertiary public hospitals.”

While this study used machine learning algorithms to assess the development status of hospital departments, the data indicators selected represent only a subset of what we believe can evaluate the workload and developmental potential of hospital departments. These indicators may have limitations, as they were chosen based on our understanding of hospital workload and departmental development. Different hospitals may have different characteristics and development needs; therefore, other hospitals should adjust these indicators appropriately based on their specific circumstances. For instance, certain specialties may require specific indicators to more accurately reflect their workload and potential for development. Additionally, as medical technology and patient needs continue to evolve, the assessment models and indicator systems will also need to be regularly updated and adjusted to ensure their relevance and effectiveness. Consequently, the model we present serves as a starting point, and we encourage future research to explore additional indicators that could enhance the model’s comprehensiveness and adaptability.

Machine learning is a promising research area for the solution of numerous societal problems, and it is rapidly gaining influence in several important teaching, research, and policy-making areas [10]. Machine learning enables the training of machines to learn certain behaviors or characteristics and then to think and make decisions based on those learned behaviors, thus enabling the imitation of human reasoning and behavior [11]. Davison [12] and Edeh et al [13] argue that machine learning-based predictions are reliable if the model is correctly validated after being trained on the data set. Machine learning models are trained using large amounts of data from research data sets, identifying trends and patterns in the data, some of which are often difficult for humans to detect. By understanding and analyzing these patterns, decision makers can gain a deeper understanding of their organization’s original work patterns and modify the direction of future guidance [14]. In this study, we applied the SVM machine learning algorithm to filter 8 important indexes, including the number of outpatient visits, surgical cases, day surgery, radiological examinations, intraoperative radiological examinations, large medical equipment examinations, as well as medical income, and total RW. Among them, outpatient visits and surgical cases represent the most fundamental indicators of hospital workload. The “Best Hospitals in America” honor roll (US News & World Report), a framework for assessing health system performance (World Health Organization), and the “World’s Best Hospitals” ranking in the United States refer to indexes of patient volume and surgical procedures [15,16].

Day surgery is important in reducing the duration of hospital stay and improving operational efficiency. Patients undergoing day surgery typically have shorter hospital stays, lower risk of infection, faster postoperative recovery, and lower costs. The global trend is the day surgery, which accounts for an increasing proportion of all procedures [17]. Day surgery does not increase the risk of complications or unplanned readmission rates for patients [18], and the percentage of day surgery in advanced hospitals worldwide is already as high as 70%-80% [19]. The higher the volume of day surgery, the faster the bed turnover, and the lower the cost to the patient. According to the DRG policies implemented globally, the higher the percentage of ambulatory surgical procedures a hospital performs each day, the more Medicare funds they receive. As a result, the “medical income” filtered by machine algorithms may increase accordingly, and the development of the discipline has sufficient funding to serve as the basis for a virtuous cycle. Furthermore, day surgery has the potential to significantly enhance patient satisfaction [19].

In this study, the number of radiological examinations, intraoperative radiological examinations, and large medical equipment examinations were filtered out using an SVM algorithm, likely due to the outstanding performance of the orthopedic departments in the study hospital. Orthopedic cases accounted for a significant majority of the total patient caseload, reaching 75.36%. Modalities such as digital radiography, computed tomography, magnetic resonance imaging, and intraoperative radiological examinations are indispensable for orthopedic inpatients. It is not surprising that medical revenue, which is the most fundamental and crucial operational metric for hospitals, was incorporated into the model. Interestingly, while the case mix index, a pivotal determinant in Medicare’s DRG reimbursement schema [20], was not selected by the algorithm, its derivative, the total RW, was. This suggests that the algorithm prioritizes a composite measure that encompasses both the complexity and the volume of diseases treated within a department. This study further developed a risk assessment model based on 8 critical indicators, delineating departments into low- and high-risk clusters. Strikingly, departments with lower development scores, as gauged by expert questionnaires, were consistently categorized within the high-risk cluster, indicating a correlation between expert perceptions and algorithmic risk stratification. In the nomogram, the department of pediatric orthopedic emerged with the lowest risk score, a finding potentially attributable to its robust disciplinary development and status as an early establishment within the hospital. It is the pioneering and largest specialized department dedicated to the treatment of skeletal deformities and injuries in children across Northwest China. The recent establishment of a pediatric orthopedic hospital within a hospital signifies a strategic move toward further subspecialty differentiation and advancement. The department of lumbar surgery achieved the highest total development score on the expert questionnaire. The department of intervertebral disc diseases and spinal deformities surgery, spinal minimally invasive surgery, cervical surgery, and spinal degenerative diseases and spinal oncologic were ranked second, third, fourth, and sixth, respectively, in the total department development score. These hospital departments were split from a department a few years ago and have since grown rapidly. The hospital-within-a-hospital spine hospital they formed is the earliest, largest, and most specialized spine hospital in Northwest China. Experts’ impressions of these departments may be related to the combined strength of the 5 departments that make up the spine hospital. In addition, the risk ranking of the nomogram and expert opinion ranking of the remaining hospital departments largely matched, and the hospital departments that ranked low in the expert questionnaire all belonged to cluster B in the consensus clustering classification, which is the high-risk cluster. Therefore, our model was accurate.

Furthermore, the robustness of our analytical approach is evidenced by the cosine similarity results, which indicate a remarkably high consistency between the 2 algorithms used in our study, with a similarity coefficient of 0.94. This level of consistency underscores the reliability of our findings and supports the validity of the risk assessment model. The Pearson correlation analysis further substantiates the significant relationship between the data sets, with a correlation coefficient of 0.734 and an exceedingly low *P* value of 4.85610^–8,^ indicating a strong and statistically significant association. This integration of statistical measures into our discussion underscores the methodological rigor of our study. The alignment of expert opinion with algorithmic risk stratification not only lends credence to the model's categorization but also highlights the use of expert insights in risk assessment. The significant correlation and high similarity between the data sets used further validated the model's efficacy in differentiating between high- and low-risk departments. These findings collectively contribute to a nuanced understanding of departmental risk, offering valuable insights for hospital administrators and policy makers in strategizing resource allocation and risk management initiatives.

The cooperation between hospital departments is not an occasional collaboration but a regular and extensive joint effort that must be patient-centered [21]. The cooperation between departments gives the overall hospital a stronger possibility to deal with all types of difficult cases and health emergencies. Professional division of labor creates conditions for in-depth research and the disciplinary development of medical science, which is an inevitable trend in the development of modern medicine. However, the human body is a unified whole, and diseases induced by a single factor constitute only a small part of the clinical picture. Even in diseases with a clear etiology, such as trauma, the cause, location, nature, and extent of the injury may result in damage to different sites and organs, often requiring assistance from different departments [22]. In Shaanxi province, where the study hospital is located, 60% of patients with orthopedic diseases are treated at the study hospital, which is a general hospital with orthopedics as the absolute leading specialty. A strong correlation was found between all departments in the study hospitals. However, ophthalmology and gynecology and obstetrics were the 2 departments least associated with this hospital’s low-risk category, and both also scored lower in the expert opinion assessment questionnaire validation data set. The consensus among the experts was that the medical treatment capacity of these 2 departments was inadequate. During hospital-wide consultations, especially in patients with multiple compound injuries, owing to the low technical capacity of the ophthalmology or gynecology and obstetrics departments, they cannot receive or manage the corresponding complications or comorbidities, and the patients will be forced to be transferred. This affects the ability of the hospital to handle critically ill patients. In future studies, we intend to develop a model for further subspecialty differentiation assessment for well-developed departments, based on data related to all diseases in that department.

### Conclusions

In summary, our machine learning algorithm provides an accurate assessment model for evaluating the coordination and development of hospital departments, The expert validation data set validates our model with a relatively good match. Combined with the actual situation of the hospital, departments with high-risk scores affect the treatment of some critically ill patients, leading to a lack of coordination in the entire hospital department’s ecological chain. Departments with low-risk scores must be considered for further subspecialty disciplinary differentiation, which is fully consistent with the work plan for those departments in the hospital that have been differentiated and will be differentiated in the future.

## Appendix

Multimedia Appendix 1 Index table of study indicators and their IDs.

Multimedia Appendix 2 Additional methods and results.

Multimedia Appendix 3 Hospital department index with corresponding IDs.

Multimedia Appendix 4 Departments risk scores of nomogram model.

Multimedia Appendix 5 Experts' rating scale for hospital departments.

## Abbreviations

DRG: diagnosis-related group
HIS: hospital information system
RW: relative weight
SVM: support vector machine

## Data Availability

The data sets generated during and/or analyzed during the current study are available from the corresponding author on reasonable request.
