# Supplementary material for: Constructing a Hospital Department Development–Level Assessment Model: Machine Learning and Expert Consultation Approach in Complex Hospital Data Environments
Source: JMIR Form Res. 2024 Sep 4;8:e54638. doi: 10.2196/54638 (PMC11411220; doi:10.2196/54638)
Supplement: Multimedia Appendix 1 [file formative_v8i1e54638_app1.docx]

**Multimedia Appendix 1**
Index table of study indicators and their IDs

| **Index ID** | **Index Name** |
| --- | --- |
| A1 | Number of discharges |
| A2 | Outpatient visits |
| A3 | Number of surgical cases |
| A4 | Number of level 4 operations |
| A5 | Number of ambulatory (day) surgery |
| A6 | Number of medical ultrasonics examination of Ultrasound Diagnostic Department I |
| A7 | Number of medical ultrasonics examination of Ultrasound Diagnostic Department II |
| A8 | Number of ueurophysiological examination |
| A9 | Number of supports used |
| A10 | Number of radiological examinations |
| A11 | Number of intraoperative radiological examinations |
| A12 | Number of pathology examinations |
| A13 | Number of Magnetic Resonance Imaging examinations |
| A14 | Number of clinical laboratory examinations |
| A15 | Number of Computed Tomography examinations |
| A16 | Number of in-hospital consultations |
| A17 | Number of blood transfusion |
| A18 | Number of bone mineral density measurements |
| A19 | Number of large medical equipment examinations |
| A20 | Dosage of bid-winning drug |
| A21 | Income from medical services |
| A22 | Medical income |
| A23 | Total pharmaceutical income |
| A24 | Income from adjuvant drugs |
| A25 | Total Relative Weights |
| A26 | Number of discharged patient files |
| A27 | Number of Diagnosis Related Groups |
| A28 | Defined Daily Doses |
| A29 | Utilization rate of essential drugs in inpatients |
| A30 | Proportion of discharged patients undergoing minimally invasive surgery |
| A31 | Percentage of discharged patients undergoing surgery |
| A32 | Positive rate of large medical equipment examination |
| A33 | Admission waiting time |
| A34 | Case-Mix Index |
