# Supplementary material for: Constructing a Hospital Department Development–Level Assessment Model: Machine Learning and Expert Consultation Approach in Complex Hospital Data Environments
Source: JMIR Form Res. 2024 Sep 4;8:e54638. doi: 10.2196/54638 (PMC11411220; doi:10.2196/54638)
Supplement: Multimedia Appendix 2 [file formative_v8i1e54638_app2.docx]

**Multimedia Appendix 2**

**Methods**

**The k-means clustering algorithm**

The k-means clustering algorithm is a maximum expectation algorithm[1] that performs iterative solution and cluster analysis in two stages: "expectation" and "maximization". This algorithm helps to discover clusters of different types of hospital departments. The algorithm divides these hospital departments into k clusters and randomly selects k departments as the initial clustering centers. Then the distance between each department and each cluster center is calculated, and each department is assigned to the cluster center closest to it.

**Support Vector Machine**

SVM is an advanced machine learning algorithm that can effectively handle nonlinear classification. It also enhances the model's generalization capabilities and computational efficiency by reducing the number of features. The SVM recursive feature elimination (SVM-RFE), a derivative algorithm of SVM, is dedicated to identifying the most critical variables from a multitude of features that contribute to the classifier's performance. Through an iterative process, SVM-RFE progressively discards features with less significant contributions to the model, ultimately refining a set of features that are both streamlined and efficient. This approach not only bolsters the predictive accuracy of the model but also optimizes computational efficiency, making the analysis process more precise and effective.[2, 3]

**Questionnaire Scoring Criteria**

Questionnaire Scoring Criteria

The hospital departments were evaluated based on two criteria: the level of departmental development and the breadth and balance. A five-point scale was utilized to assess the developmental level of hospital departments. The scale was defined as follows: 1 point = "the level of county medical institutions that can only treat basic and general diseases"; 2 points = "municipal medical level, treating mostly common and basic illnesses"; 3 points = "provincial medical level, treating mainly high incidence diseases and some difficult diseases"; 4 points = "district medical center level, treating mostly difficult diseases"; 5 points = "national medical center level, treating diseases that are mostly rare or uncommon". The breadth and balance dimension was also scored on a five-point scale as follows: 1 points = "The department can treat 0-20% of patients with related diseases and can rarely collaborate with other departments to treat patients with complex diseases"; 2 points = "The department can treat 20-40% of patients with related diseases and rarely collaborates with other departments to treat patients with complex diseases"; 3 points = "The department can treat 40-60% of patients with related diseases and can collaborate with other departments to treat 40-60% of patients with complex diseases"; 4 points = "The department can treat 60-80% of patients with related diseases and can collaborate with other departments to treat 60-80% of patients with complex diseases, with less collaboration with other departments"; 5 point = "The department can treat 80-100% of patients with related diseases and can collaborate strongly with other departments to treat 80-100% of patients with complex diseases". The median weights of the two categories of indexes were calculated based on the expert questionnaire. These categories were (1) the level of sectoral development and (2) breadth and balance. The weightings were 60% and 40%, respectively. The score of each expert in each department was determined according to the weighting. The overall rating for department development was the sum of the ratings of the experts in that department. The departments were then ranked based on the total department development score.

**Results**

**Consensus Clustering Analysis Details**

The item-consensus histogram (**Appendix figure 1a-h**) illustrates the degree of agreement among cluster assignments for each department. The average clustering consensus score was higher for k=2 (**Appendix figure 1i**), indicating that the classification results of this subcluster were more stable than the others. The matrix heatmap (**Fig. 2a-h**) visually represents the separation quality between clusters, with warmer colors indicating higher correlation within clusters and cooler colors suggesting lower correlation between clusters. The cumulative distribution function (CDF) curve (**Fig. 2i**) and its delta area scores (**Fig. 2j**) offer a quantitative assessment of the clustering quality, with the minimal slope indicating optimal clustering at k=2. The trace plot (**Fig. 2k**) confirms the stability of the classification at k=2.


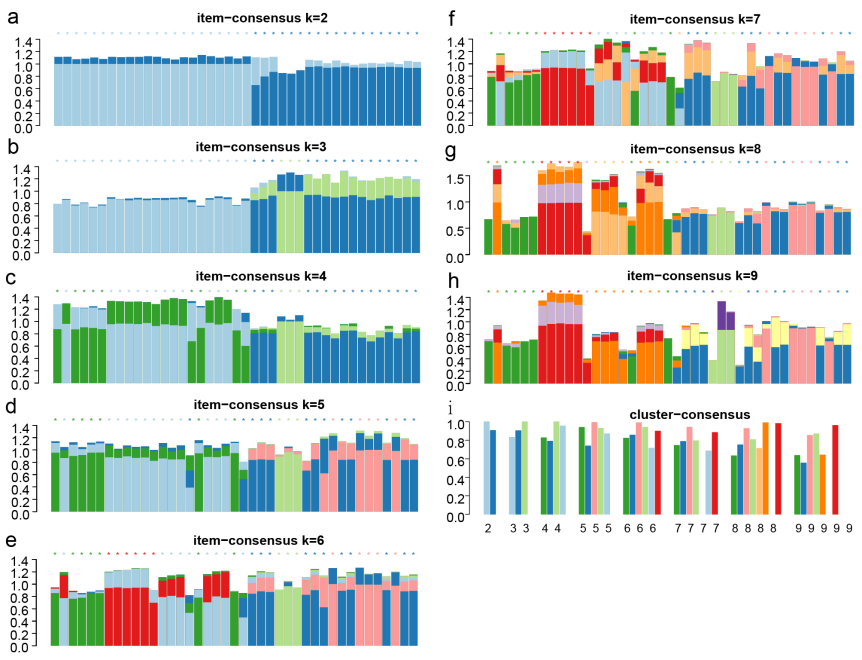


**Appendix figure 1** Consensus cluster analysis of hospital department indexes. a-h: Item-Consensus Plot. The ordinate represents item-consensus values. When the k value is different, each sample will have an item-consensus value corresponding to a different cluster. The vertical bar represents each sample, and the height of the vertical bar represents the total item-consensus values for that sample. There is a small cross above each sample, and the color of the small cross indicates the cluster into which the sample is divided. i: Cluster-Consensus Plot. This figure shows the cluster-consensus value (mean value of pairwise consensus values of members in the cluster) for each category under different k values. Higher (lower) values represent higher (lower) stability. It can be used to determine the level of cluster-consensus values under the same k value and between different k values.

**References**

1. Yang J, Wang Y, Yao X, Lin C. Adaptive Initialization Method for K-Means Algorithm. Frontiers in artificial intelligence. 2021;4:740817. PMID: 34901837. doi: 10.3389/frai.2021.740817.

2. FuFeng L, Changbo Z, Zheng X, Yiqin W, Xiaobo Z, Guo-Zheng L. Computer-assisted lip diagnosis on Traditional Chinese Medicine using multi-class support vector machines. BMC Complement Altern Med. 2012;12(0). PMID: 22898352. doi: 10.1186/1472-6882-12-127.

3. Hector S, Clarissa V, Esteban V, Josep M O, Ferran R. SVM-RFE: selection and visualization of the most relevant features through non-linear kernels. BMC Bioinformatics. 2018;19(1). PMID: 30453885. doi: 10.1186/s12859-018-2451-4.
