# Supplementary material for: Constructing a Hospital Department Development–Level Assessment Model: Machine Learning and Expert Consultation Approach in Complex Hospital Data Environments
Source: JMIR Form Res. 2024 Sep 4;8:e54638. doi: 10.2196/54638 (PMC11411220; doi:10.2196/54638)
Supplement: Multimedia Appendix 3 [file formative_v8i1e54638_app3.docx]

**Multimedia Appendix 3**

Hospital department index with corresponding IDs.

| Department ID | Department |
| --- | --- |
| B1 | Department of Pediatric Orthopedic |
| B2 | Department of Ear, Nose & Throat and Head & Neck-usually and Plastic Surgery |
| B3 | Department of Rheumatology Immunology and Endocrinology |
| B4 | Department of Gynecology and Obstetrics |
| B5 | Department of Orthopaedic Oncology |
| B6 | Department of Peri-pelvic Traumatic Orthopaedic |
| B7 | Department of Upper Extremity Traumatic Orthopaedic |
| B8 | Department of Lower Extremity Traumatic Orthopaedic |
| B9 | Department of Orthopaedic Microsurgery |
| B10 | Department of Rheumatic and Immune-related Orthopedic Joint Surgery |
| B11 | Department of Osteonecrosis and Joint Reconstruction surgery |
| B12 | Department of Hip Joint Surgery |
| B13 | Department of Knee Joint Surgery |
| B14 | Department of Respiratory Diseases |
| B15 | Department of Emergency |
| B16 | Department of Cervical Surgery |
| B17 | Department of Spinal Degenerative Diseases and Spinal Oncologic |
| B18 | Department of Spinal Minimally Invasive Surgery |
| B19 | Department of Lumbar Surgery |
| B20 | Department of Intervertebral Disc Diseases and Spinal Deformities Surgery |
| B21 | Department of Bone and Joint Rehabilitation |
| B22 | Department of Neurospinal Rehabilitation |
| B23 | Department of Pain Intervention |
| B24 | Department of Urology Surgery |
| B25 | Department of General Practice |
| B26 | Department of Neurology |
| B27 | Department of Neurosurgery |
| B28 | Hand Surgery Center II |
| B29 | Hand Surgery Center I |
| B30 | Department of Gastroenterology |
| B31 | Department of Digestive Surgery |
| B32 | Department of Cardiovascular Medicine |
| B33 | Department of Thoracic Surgery |
| B34 | Department of Hematology and Oncology |
| B35 | Department of Ophthalmology |
| B36 | Department of Shoulder and Elbow Surgery |
| B37 | Department of Knee and Ankle Surgery |
| B38 | Department of Integrated Traditional Chinese Medicine and Western Medicine Orthopedics |
| B39 | Department of Critical Care Medicine |
| B40 | Department of Peripheral Vascular Medicine |
| B41 | Department of Foot and Ankle Surgery |
