# Supplementary material for: Constructing a Hospital Department Development–Level Assessment Model: Machine Learning and Expert Consultation Approach in Complex Hospital Data Environments
Source: JMIR Form Res. 2024 Sep 4;8:e54638. doi: 10.2196/54638 (PMC11411220; doi:10.2196/54638)
Supplement: Multimedia Appendix 4 [file formative_v8i1e54638_app4.docx]

**Multimedia Appendix 4**

Departments risk scores of nomogram model

| Department | Type | Points |
| --- | --- | --- |
| Department of Pediatric Orthopedic | A | 127.41 |
| Department of Foot and Ankle Surgery | A | 128.85 |
| Hand Surgery Center I | A | 134.43 |
| Department of Lower Extremity Traumatic Orthopaedic | A | 146.45 |
| Hand Surgery Center II | A | 146.89 |
| Department of Spinal Degenerative Diseases and Spinal Oncologic | A | 148.73 |
| Department of Integrated Traditional Chinese Medicine and Western Medicine Orthopedics | A | 150.00 |
| Department of Rheumatic and Immune-related Orthopedic Joint Surgery | A | 151.10 |
| Department of Intervertebral Disc Diseases and Spinal Deformities Surgery | A | 152.18 |
| Department of Peri-pelvic Traumatic Orthopaedic | A | 153.04 |
| Department of Lumbar Surgery | A | 153.14 |
| Department of Hip Joint Surgery | A | 153.18 |
| Department of Upper Extremity Traumatic Orthopaedic | A | 153.62 |
| Department of Shoulder and Elbow Surgery | A | 154.88 |
| Department of Cervical Surgery | A | 155.21 |
| Department of Osteonecrosis and Joint Reconstruction surgery | A | 155.32 |
| Department of Spinal Minimally Invasive Surgery | A | 155.47 |
| Department of Knee Joint Surgery | A | 155.96 |
| Department of Orthopaedic Microsurgery | A | 156.63 |
| Department of Orthopaedic Oncology | A | 158.85 |
| Department of Knee and Ankle Surgery | A | 158.93 |
| Department of Emergency | A | 160.21 |
| Department of Thoracic Surgery | B | 185.49 |
| Department of Respiratory Diseases | B | 185.83 |
| Department of Digestive Surgery | B | 186.37 |
| Department of Peripheral Vascular Medicine | B | 187.61 |
| Department of General Practice | B | 188.22 |
| Department of Ear, Nose & Throat and Head & Neck-usually and Plastic Surgery | B | 189.31 |
| Department of Urology Surgery | B | 189.55 |
| Department of Rheumatology Immunology and Endocrinology | B | 191.26 |
| Department of Pain Intervention | B | 191.34 |
| Department of Cardiovascular Medicine | B | 191.35 |
| Department of Bone and Joint Rehabilitation | B | 192.98 |
| Department of Neurology | B | 193.22 |
| Department of Neurosurgery | B | 197.48 |
| Department of Neurospinal Rehabilitation | B | 198.90 |
| Department of Gynecology and Obstetrics | B | 199.71 |
| Department of Ophthalmology | B | 204.98 |
| Department of Gastroenterology | B | 213.67 |
| Department of Hematology and Oncology | B | 214.46 |
| Department of Critical Care Medicine | B | 227.01 |
