# Supplementary material for: Constructing a Hospital Department Development–Level Assessment Model: Machine Learning and Expert Consultation Approach in Complex Hospital Data Environments
Source: JMIR Form Res. 2024 Sep 4;8:e54638. doi: 10.2196/54638 (PMC11411220; doi:10.2196/54638)
Supplement: Multimedia Appendix 5 [file formative_v8i1e54638_app5.docx]

**Multimedia Appendix 5**

Experts' rating scale for hospital departments

| Department | Grade |
| --- | --- |
| Department of Lumbar Surgery | 155.8 |
| Department of Intervertebral Disc Diseases and Spinal Deformities Surgery | 155.6 |
| Department of Spinal Minimally Invasive Surgery | 155.0 |
| Department of Cervical Surgery | 153.2 |
| Department of Osteonecrosis and Joint Reconstruction surgery | 153.0 |
| Department of Spinal Degenerative Diseases and Spinal Oncologic | 152.4 |
| Department of Knee Joint Surgery | 151.6 |
| Department of Hip Joint Surgery | 151.4 |
| Department of Knee and Ankle Surgery | 149.2 |
| Department of Peri-pelvic Traumatic Orthopaedic | 148.8 |
| Department of Foot and Ankle Surgery | 148.4 |
| Department of Lower Extremity Traumatic Orthopaedic | 146.4 |
| Hand Surgery Center I | 146.4 |
| Department of Pediatric Orthopedic | 143.8 |
| Department of Upper Extremity Traumatic Orthopaedic | 143.0 |
| Hand Surgery Center II | 142.8 |
| Department of Shoulder and Elbow Surgery | 141.8 |
| Department of Orthopaedic Microsurgery | 140.2 |
| Department of Rheumatic and Immune-related Orthopedic Joint Surgery | 136.6 |
| Department of Integrated Traditional Chinese Medicine and Western Medicine Orthopedics | 135.4 |
| Department of Bone and Joint Rehabilitation | 124.2 |
| Department of Neurospinal Rehabilitation | 123.2 |
| Department of Gastroenterology | 122.8 |
| Department of Pain Intervention | 120.6 |
| Department of Digestive Surgery | 117.0 |
| Department of Orthopaedic Oncology | 114.6 |
| Department of Peripheral Vascular Medicine | 114.0 |
| Department of Cardiovascular Medicine | 110.0 |
| Department of Respiratory Diseases | 110.0 |
| Department of Rheumatology Immunology and Endocrinology | 108.4 |
| Department of Critical Care Medicine | 108.0 |
| Department of Ear, Nose & Throat and Head & Neck-usually and Plastic Surgery | 106.8 |
| Department of Emergency | 106.8 |
| Department of Neurosurgery | 106.6 |
| Department of Thoracic Surgery | 103.2 |
| Department of General Practice | 101.8 |
| Department of Neurology | 101.8 |
| Department of Urology Surgery | 100.2 |
| Department of Hematology and Oncology | 98.0 |
| Department of Ophthalmology | 78.4 |
| Department of Gynecology and Obstetrics | 62.0 |
